# Acetylcholinesterase Inhibition and Antioxidant Activity of *N*-*trans*-Caffeoyldopamine and *N*-*trans*-Feruloyldopamine

**DOI:** 10.3390/scipharm86020011

**Published:** 2018-04-04

**Authors:** Muamer Dizdar, Danijela Vidic, Franc Požgan, Bogdan Štefane, Milka Maksimović

**Affiliations:** 1 Faculty of Science, University of Sarajevo, Zmaja od Bosne 33-35, 71000 Sarajevo, Bosnia and Herzegovina; muamer.dizdar@pmf.unsa.ba (M.D.); danijela.vidic@gmail.com (D.V.); 2 Faculty of Chemistry and Chemical Technology, University of Ljubljana, Večna pot 113, 1000 Ljubljana, Slovenia; Franc.Pozgan@fkkt.uni-lj.si (F.P.); Bogdan.Stefane@fkkt.uni-lj.si (B.Š.)

**Keywords:** acetylcholinesterase inhibition, antioxidant activity, *N*-*trans*-feruloyldopamine, *N*-*trans*-caffeoyldopamine

## Abstract

Phenolic acids and their derivatives found in nature are well-known for their potential biological activity. In this study, two amides derived from *trans*-caffeic/ferulic acid and dopamine were synthesized and characterized by Fourier-transform infrared spectroscopy (FTIR), mass spectrometry, proton and carbon-13 nuclear magnetic resonance spectroscopy. The compounds were tested for the inhibition of acetylcholinesterase (AChE) from *Electrophorus electricus* and for antioxidant activity by scavenging 2,2-diphenyl-1-pycrylhydrazyl free radical (DPPH^•^) and 2,2′-azinobis(3-ethylbenzothiazoline-6-sulphonic acid) radical cation (ABTS^•+^), reducing ferric ions, and ferrous ions chelation. *N*-*trans*-Feruloyldopamine displayed the highest inhibitory effect on AChE with half-maximal inhibitory concentration (*IC*_50_) values of 8.52 μM. In addition, an in silico study was done to determine the most favorable AChE cluster with the synthesized compounds. Further, these clusters were investigated for binding positions at the lowest free binding energy. Both synthesized hydroxycinnamates were found to be better antioxidants than the parent acids in in vitro tests applied. *N*-*trans*-Caffeoyldopamine showed the best antioxidant activity in the three tested methods—against non-biological stable free radicals *IC*_50_ 5.95 μM for DPPH^•^, 0.24 μM for the ABTS^•+^ method, and for reducing power (ascorbic acid equivalent *(AAE)* 822.45 μmol/mmol)—while for chelation activity against Fe^2+^ ions *N*-*trans*-feruloyldopamine had slightly better antioxidant activity (*IC*_50_ 3.17 mM).

## 1. Introduction

Phenolic acids constitute a family of natural compounds that can be found in a wide variety of plants and food [1]. The biological activity of phenolic acids and other phenolic compounds has attracted much attention and has been studied by many research groups in recent years. The compounds are known to have acetylcholinesterase (AChE)-inhibitory [2], antibacterial [3], anti-inflammatory [4], antioxidant [5,6], antiviral [7], and immunomodulatory [8] properties. These properties are associated with either their properties as antioxidants and enzyme inhibitors or their binding activity with specific receptors. Also, derivatives of phenolic acids, which primarily include esters, ethers, glycosides, and amides, were tested and showed various biological activities [9,10,11]. Some of these derivatives belong to clovamide type derivatives that can be found in nature [11,12] but have also been synthesized [13]. This group of derivatives includes *N*-*trans*-caffeoyldopamine and *N*-*trans*-feruloyldopamine, which has been shown to have potent protective effects against hepatotoxicity [14], are β2-adrenoceptor agonists [15], and inhibit platelet–leukocyte interactions and lipid peroxidation [12,16]. All these findings make *N*-*trans*-caffeoyldopamine and *N*-*trans*-feruloyldopamine interesting bioactive compounds for pharmaceutical and food research as potentially useful ingredients of some medicines. According to our best knowledge, a comprehensive study of AChE inhibition and the antioxidant activity of these clovamide type derivatives has not been reported so far.

Acetylcholinesterase is widely distributed in the central and peripheral nervous systems where it plays a critical role in quickly hydrolyzing the neurotransmitter acetylcholine, which is involved in the pathogenesis of Alzheimer’s disease (AD). Hydrolysis is the result of nucleophilic attack of the carbonyl carbon by a serine residue in the enzyme active center and involves charge transfer via a histidine residue [17]. However, there are several subsites, which can be distinguished in the AChE active site: esteratic subsite (also called the catalytic triad), oxanion hole, anionic subsite, acyl binding pocket, peripheral anionic subsite, and other residues of the omega loop [18]. Recognized factors in AD development include free radicals, inflammation of the brain tissue, and acetylcholine deficiency. Therefore, acetylcholine breakdown in the brain can be prevented by the inhibition of AChE activity, leading to an increase in acetylcholine concentration [19]. Today, several AChE inhibitors are used to treat AD, including tacrine, donepezil, rivastigmine, and carbamates. Also, some natural products show the ability to inhibit AChE, and the largest number of studies include alkaloids [20], phenolic compounds [2,21], and terpenes [22].

Free radicals derived from oxygen, nitrogen, and sulfur compounds are highly reactive molecules because of the presence of an unpaired electron, which is formed in metabolic processes within the cell. Their role in signaling, apoptosis, gene expression, and ion transport has been proven [23]. In addition, they can attack purine and pyrimidine bases, amino acids, and unsaturated fatty acids, causing oxidative stress and damage to DNA, RNA, proteins, carbohydrates, and lipids. Many studies have demonstrated the correlation between these processes and a large number of diseases [24]. Although the human body has a number of enzymatic systems that protect it from oxidative stress, the ability of these systems decreases with aging, increasing the need to take in antioxidants from dietary supplements [25]. A large number of compounds from dietary supplements exhibit antioxidant activity in living systems, among which the most important are l(+)-ascorbic acid, carotenoids, vitamin E, and phenolic compounds [26].

Following the molecular combination strategy, the aim of this study was to provide a full analysis of the structure-AChE inhibition/antioxidant activity relationship for two synthesized *trans*-hydroxycinnamic amide derivatives. These compounds were investigated for their ability to inhibit the AChE from *Electrophorus electricus* by a spectrophotometrically assay based on an Ellman method [27]. Also, molecular docking studies of AChE were done to estimate the binding positions and affinity of the complex target/ligand. Their efficiency as radical scavengers was evaluated by their reactivity toward the stable 2,2-diphenyl-1-pycrylhydrazyl free radical (DPPH^•^) and 2,2′-azinobis(3-ethylbenzothiazoline-6-sulphonic acid) radical cation (ABTS^•+^), while the reducing power and chelation ability were determined by the ferric-reducing antioxidant power (FRAP) assay and the inhibition of the Fe^2+^–ferrozine complex formation.

## 2. Materials and Methods

### 2.1. Chemicals and General Tehniques

All chemicals were purchased from commercial sources and when necessary, purified following the guidelines of Armarego and Chai [28]. The monitoring of the reaction and purity of the obtained compounds was done by thin-layer chromatography (TLC) on silica gel 60 F_254_, while the column chromatography was performed on silica gel (100–200 mesh). The visualization of the chromatograms was performed either under ultraviolet light and/or with 5% FeCl_3_. The melting points were determined using a hot stage apparatus and are uncorrected. Infrared spectra were collected as potassium bromide (KBr) pellets in the 4000–400 cm^−1^ region with a PerkinElmer BX Fourier-transform infrared spectrometer (FTIR) (PerkinElmer, Waltham, MA, USA). The peak intensities are specified as strong (s), medium (m), or broad (br). The proton and carbon-13 nuclear magnetic resonance (^1^H- and ^13^C-NMR) spectra were recorded using a Bruker AVANCE Ultrashield 500 plus (Bruker, Billerica, MA, USA), using deuterated dimethyl sulfoxide (DMSO-*d*_6_) as the solvent. All shifts are given in ppm (*δ*) relative to tetramethylsilane and calibrated by the residual proton signal of DMSO-*d*_6_. The coupling constants (*J* values) are expressed in Hz. High-resolution mass spectra (MS) were obtained by the Agilent 6224 TOF mass spectrometer (Agilent Technologies, Santa Clara, CA, USA). All spectrophotometric measurements were made on a PerkinElmer Lambda 25 UV–Vis spectrophotometer (PerkinElmer, Norwalk, MA, USA).

### 2.2. Synthesis

Triethylamine (0.28 mL, 2 mmol) was added to a stirring solution of *trans*-hydoxycinnamic acid (2 mmol) dissolved in dimethylformamide (10 mL). The solution was cooled in an ice water bath and 2 mmol of dopamine hydrochloride was added followed by a solution of 2 mmol of (benzotriazol-1-yloxa)tris(dimethylamino)phosphonium hexafluorophosphate (BOP) in 5 mL dichloromethane. The mixture was stirred at 0 °C for 30 min and then at room temperature overnight. Dichloromethane was removed under reduced pressure and the resulting mixture was diluted with 100 mL water and extracted with ethyl acetate (EtOAc) (3 × 30 mL). The combined organic layers were washed with HCl (3 × 15 mL, 0.5 M), NaHCO_3_ (3 × 15 mL, 0.5 M), water (3 × 15 mL), and dried (MgSO_4_) and evaporated under reduced pressure. The mixture was then absorbed onto SiO_2_ (≈1 g, 100–200 mesh) and purified by column chromatography (EtOAc–hexane, 1:2→2:1) to give the crude products.

### 2.3. Acetylcholinesterase Inhibition

#### 2.3.1. In Vitro Assay

The AChE inhibition of tested compounds was determined by a slightly modified Ellman’s method [27], in which thiocholine produced by the action of AChE forms a yellow color with Ellman’s reagent (DTNB). Galantamine was used as a reference compound. The tested compounds were dissolved in DMSO to provide a final concentration range of 0–50 μM in the mixture solution. Briefly, 0.3 mL of 100 mM sodium phosphate buffer pH 8, 0.3 mL of sample, and 0.3 mL AChE solution containing 0.54 U/mL were mixed in a 3 mL cuvette and allowed to incubate for 15 min at 37 °C. Subsequently, 0.3 mL of a solution of acetylcholine iodide (15 mM, dissolved in water) and 1.5 mL of 3 mM DTNB were added. The absorbance at 405 nm was read during the first 5 min of the reaction. A control reaction, which was considered to have 100% activity, was carried out using the same volume of DMSO instead of tested solutions. The percentage of AChE inhibition is calculated based on the absorbance value as follows:

(1) $$\%\;\mathrm{Inhibition}\;=\;\left(1\;-\;\frac{A_{t}}{A_{0}}\right)\;\times \;100$$

where *A*_0_ is the absorbance of the control and *A*_t_ is the absorbance of the tested compound. A linear equation indicating the correlation between the common logarithm of the compound concentration and the percentage of AChE inhibition was built, from which the *IC*_50_ values (the concentration that inhibits 50% of AChE activity) of the studied compounds were extrapolated.

#### 2.3.2. Molecular Docking Studies

A molecular docking study was performed in order to suggest a possible binding mode of synthesized compounds with AChE. A SwissDock server was used for in silico prediction of the lowest free binding energy using EADock DSS software [29,30]. The crystal structure of the target AChE from *E. electricus* (1C2B) [31] was taken in protein data bank (PDB) format and analyzed in mol2 format as required for calculation. UCSF Chimera 1.12 software was used for the visualization of the results and creating three-dimensional (3D) images [32].

### 2.4. Antioxidant Activity

#### 2.4.1. 2,2-Diphenyl-1-Pycrylhydrazyl Free Radical Scavenging Assay

The 2,2-diphenyl-1-pycrylhydrazyl free radical (DPPH^•^) scavenging method is widely used to evaluate the free radical scavenging ability of natural antioxidants [33]. 2,2-Diphenyl-1-pycrylhydrazyl is a stable nitrogen centered free radical, which has a violet color that changes to yellow after reduction by the process of either hydrogen atom or single electron transfer mechanism. Compounds that are able to execute this reaction can be considered antioxidants and therefore radical scavengers [1]. In the radical form, the DPPH molecule has an absorbance of 517 nm, which disappears after the acceptance of an electron or hydrogen radical from an antioxidant compound, to become a stable diamagnetic molecule [34]. The DPPH^•^ scavenging activities of the tested compounds were estimated using the method of [35] with slight modification. To 1 mL methanolic solution of DPPH^•^ (0.08 mM), 0.1 mL of the tested compounds were added. The resulting mixture was shaken thoroughly and allowed to stand at room temperature in the dark for 30 min, after which the absorbance of the solution was measured at 517 nm. l(+)-Ascorbic acid was used as the positive control. The negative control contained DPPH^•^ and methanol. The ability to scavenge DPPH radical was calculated using the equation:

(2) $$\%\;\mathrm{DPPH}\;\mathrm{radical}\;\mathrm{scavenge}\;=\;\left(1\;-\;\frac{A_{t}}{A_{0}}\right)\;\times \;100$$

where *A*_0_ is the absorbance of the control and *A*_t_ is the absorbance of the tested compound. The DPPH^•^ absorbance decreases with an increase in the DPPH radical scavenging activity. The results were expressed as *IC*_50_ concentration, where 50% inhibition of the DPPH radical was obtained.

#### 2.4.2. 2,2′-Azinobis(3-Ethylbenzothiazoline-6-Sulphonic Acid) Radical Cation Scavenging Assay

The 2,2′-azinobis(3-ethylbenzothiazoline-6-sulphonic acid) radical cation (ABTS^•+^), which absorbs at 734 nm, is formed by oxidation of the commercially available neutral species. This oxidation can be done by potassium persulphate, manganese (IV) oxide, or electrochemical methods. In the presence of a hydrogen-donating antioxidant, the nitrogen quenches the hydrogen atom, which is followed by decolorization [36,37]. In this study, the ABTS^•+^ was produced by mixing 7 mM ABTS stock solution with 2.45 mM potassium persulfate and allowing the mixture to stand in a dark at room temperature for 16 h before use. Different concentrations of the tested compounds (0.1 mL) were added to 1 mL ABTS^•+^ solution. The reaction mixtures were mixed thoroughly, incubated at room temperature for 7 min, and the absorbance was recorded at 734 nm. The ability to scavenge the ABTS radical cation was calculated using the following equation:

(3) $$\%\;\mathrm{ABTS}\;\mathrm{radical}\;\mathrm{cation}\;\mathrm{scavenge}\;=\;\left(1\;-\;\frac{A_{t}}{A_{0}}\right)\;\times \;100$$

where *A*_0_ is the absorbance of the control and *A*_t_ is the absorbance of the tested compound. The ABTS^•+^ solution without any sample was used as a control, prepared by using the same procedure. From the percentage of the scavenging activity at different tested compounds concentrations, *IC*_50_ values were calculated and compared with those of standard l(+)-ascorbic acid solution.

#### 2.4.3. Ferric-Reducing Antioxidant Power Assay

The FRAP assay is a typical electron transfer-based method that measures the reduction of (Fe^3+^)-ligand complex to the intensely blue-colored ferrous (Fe^2+^)-ligand complex by antioxidants [38]. The FRAP assay is carried out under acidic pH conditions in order to maintain iron solubility and more importantly, drive electron transfer. This will increase the redox potential, causing a shift in the dominant reaction mechanism [39]. The FRAP assay was done according to the method described by Benzie and Strain [40] with slightly modifications. The stock solutions included 300 mM acetate buffer (pH 3.6), 10 mM 2,4,6-tris(2-pyridyl)-*s*-triazine (TPTZ) solution in ethanol, and 20 mM FeCl_3_ solution in 20 mM HCl. To prepare the FRAP working solution, acetate buffer, TPTZ, and FeCl_3_ solutions were mixed in this order at a volume ratio of 10:1:1 and then warmed at 37 °C before using. An aliquot of 0.1 mL of the tested compound was allowed to react with 3.0 mL of the FRAP working solution and 0.3 mL of water. All readings where then taken at 593 nm against a reagent blank at the end of 6 min. The results are expressed as l(+)-ascorbic acid equivalent (*AAE*).

#### 2.4.4. Fe(II)-Chelating Assay

Among the transition metals, Fe^2+^ ions are well-known for their significant role as a prooxidants in the lipid oxidation process and the formation of hydroxyl radicals in Fenton-type reactions [41]. However, effective chelating agents can reduce the damage resulting from these reactions. The most important spectrophotometric method for studying chelation activity is the inhibition of the Fe^2+^–ferrozine complex formation, during which ferrozine reacts quantitatively with Fe^2+^ ions in the presence of a chelating agent and complex formation is disrupted. Measurement of color reduction therefore allows estimation of the metal chelating activity of the coexisting chelator. The chelating abilities on Fe^2+^ by the tested compounds were estimated by the method as described by Dinis [42]. Briefly, 0.4 mL of the tested compound was added to 0.05 mL of FeCl_2_ (2 mM) solution. The reaction was initiated by the addition of ferrozine (0.2 mL, 5 mM). The total volume of the systems was adjusted to 4 mL with ethanol. Then, the mixture was shaken vigorously and left at room temperature for 10 min. The absorbance of the solution was then measured spectrophotometrically at 562 nm. The percentage of chelating effect was calculated by using the equation below:

(4) $$\%\;\mathrm{Chelating}\;\mathrm{effect}\;=\;\left(1\;-\;\frac{A_{t}}{A_{0}}\right)\;\times \;100$$

where *A*_0_ is the absorbance of the control and *A*_t_ is the absorbance of the tested compound. The results were expressed as *IC*_50_ concentration, where 50% inhibition of the Fe^2+^–ferrozine complex was obtained and compared with those of the standard disodium ethylenediaminetetraacetate (Na_2_EDTA) solution.

### 2.5. Statistical Analysis

All data on AChE inhibition/antioxidant activity are the average of triplicate analyses (*n* = 3) and the data were recorded as mean ± standard deviation. Regression analysis was performed to calculate the dose-response relation and *IC*_50_ values were obtained from the equations *y* = *a* ± *bx*.

## 3. Results and Discussion

### 3.1. Synthesis

**Figure 1:**
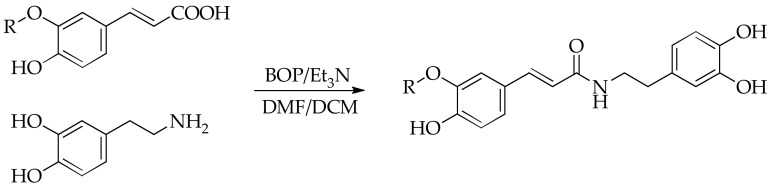
Synthesis of amide derivatives. R = H and CH_3_, respectively, for *trans*-caffeic and ferulic acid. BOP: (benzotriazol-1-yloxa)tris(dimethylamino)phosphonium hexafluorophosphate; DMF: dimethylformamide; DCM: dichloromethane; Et_3_N: triethylamine.

The synthesis of amides via reactive esters is one of the most common synthetic methods used in the chemicals of natural products and peptides. These methods are usually simple, require minimal purification, and give high yields [43]. For the synthesis of amide derivatives, a coupling method with BOP was used (Figure 1) [44]. 

Both synthesized derivatives are characterized by several instrumental methods and the obtained results were related to the proposed structures. All corresponding spectra (MS, ^1^H and ^13^C-NMR are given in the Supplementary Materials.

#### 3.1.1. *N*-*trans*-Caffeoyldopamine

Yield: 258 mg, 41%; Beige solid; *R*_f_ 0.24 (EtOAc–hexane, 1:1); Melting point 182–184 °C; *ν*/cm^−1^ (KBr): 3450–3250 (br, s), 1653 (s), 1600 (s), 1521 (s), 1443 (s), 1372 (m), 1283 (s), 1195 (s), 1113 (s); ^1^H-NMR (500 MHz, DMSO-*d*_6_) *δ*_H_: 9.34 (s, 1H), 9.11 (s, 1H), 8.75 (s, 1H), 8.64 (s, 1H), 8.01 (t, *J* = 5.7 Hz, 1H), 7.23 (d, *J* = 15.7 Hz, 1H), 6.94 (d, *J* = 2.1 Hz, 1H), 6.83 (dd, *J* = 8.2, 2.1 Hz, 1H), 6.74 (d, *J* = 8.1 Hz, 1H), 6.64 (d, *J* = 7.9 Hz, 1H), 6.60 (d, *J* = 2.1 Hz, 1H), 6.46 (dd, *J* = 8.1, 2.1 Hz, 1H), 6.32 (d, *J* = 15.7 Hz, 1H), 3.30 (q, *J* = 7.0 Hz, 2H), 2.57 (t, *J* = 7.5 Hz, 2H); ^13^C-NMR (125 MHz, DMSO-*d*_6_) *δ*_C_: 165.75, 147.71, 145.99, 145.52, 144.00, 139.40, 130.74, 126.89, 120.83, 119.68, 119.08, 116.45, 116.21, 115.96, 114.26, 41.19, 35.23; HRMS (ESI+, *m*/*z*): 316.1179 [M + H]^+^ (C_17_H_18_NO_5_ requires 316.1177).

#### 3.1.2. *N*-*trans*-Feruloyldopamine

Yield: 349 mg, 53%; Pale yellow solid; *R*_f_ 0.32 (EtOAc–hexane, 1:1); Melting point 144–145 °C; *ν*/cm^−1^ (KBr): 3450–3250 (br, s), 1654 (s), 1594 (s), 1516 (s), 1448 (m), 1374 (m), 1272 (s), 1210 (m), 1160 (m), 1123 (m); ^1^H-NMR (500 MHz, DMSO-*d*_6_) *δ*_H_: 9.41 (s, 1H), 8.75 (s, 1H), 8.64 (s, 1H), 7.97 (t, *J* = 5.7 Hz, 1H), 7.31 (d, *J* = 15.6 Hz, 1H), 7.11 (d, *J* = 2.0 Hz, 1H), 6.98 (dd, *J* = 8.2, 2.0 Hz, 1H), 6.79 (d, *J* = 8.1 Hz, 1H), 6.64 (d, *J* = 7.9 Hz, 1H), 6.60 (d, *J* = 2.1 Hz, 1H), 6.46 (dd, *J* = 8.0, 2.1 Hz, 1H), 6.43 (d, *J* = 15.7 Hz, 1H), 3.80 (s, 3H), 3.31 (q, *J* = 6.9 Hz, 2H), 2.58 (t, *J* = 7.4 Hz, 2H); ^13^C-NMR (125 MHz, DMSO-*d*_6_) *δ*_C_: 165.74, 148.68, 148.28, 145.52, 143.99, 139.30, 130.71, 126.91, 121.96, 119.67, 119.54, 116.44, 116.11, 115.96, 111.20, 55.98, 41.15, 35.18; HRMS (ESI+, *m*/*z*): 330.1336 [M + H]^+^ (C_18_H_20_NO_5_ requires 330.1340).

### 3.2. Acetylcholinesterase Inhibition

Acetylcholinesterase inhibitors have been widely used in the treatment of neurodegenerative disease. Both synthesized derivatives and phenolic acids were tested to evaluate their ability to inhibit AChE from *E. electricus*. The results are reported in Table 1. 

Among the tested compounds, *N*-*trans*-feruloyldopamine showed the best ability to inhibit AChE with *IC*_50_ 8.52 μM, while the weakest inhibitory activity was found in the case of caffeic acid (*IC*_50_ 42.81 μM). The reported data clearly indicate that two main features influence the enzyme inhibition:

- the nature of the R substituent at the aromatic ring, which plays a key role in the inhibitory potency, and
- the presence of the dopamine moiety via the amide-coupled bond.

**Figure 2:**
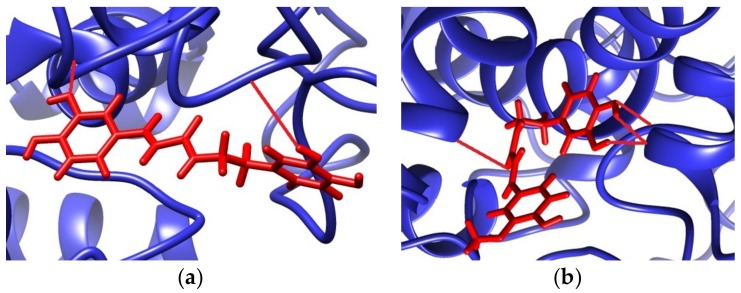
The complex target–ligand as viewed in UCSF Chimera 1.12. Possible interaction of AChE from *E. electricus* with: (**a**) *N*-*trans*-caffeoyldopamine; (**b**) *N*-*trans*-feruloyldopamine.

To support the experimental results obtained by enzymatic inhibition, the interaction between AChE and tested compounds was also predicted by molecular docking using SwissDock (freely available on Swiss Institute of Bioinformatics Website) [29]. The 3D crystal structure of AChE was obtained from the Research Collaboratory for Structural Bioinformatics (RCSB) Protein Data Bank (PDB ID: 1C2B) [31]. The protein preparation and the molecular docking results visualization were carried out using the UCSF Chimera 1.12 package. The docking results predicted that the lowest binding energy (ΔG) was equal to −34.12 kJ/mol for *N*-*trans*-caffeoyldopamine and −36.13 kJ/mol for *N*-*trans*-feruloyldopamine. The more negative value of ΔG indicated good binding affinity of the target molecule with the ligand and was correlated with the number of hydrogen bonds in the resulting cluster. In our case, the estimated ΔG corresponded to the interaction with the peripheral anionic, esteratic subunit (catalytic triad), and anionic subsite (Figure 2). 

For *N*-*trans*-caffeoyldopamine, the predicting binding positions included a hydrogen bond between the *m*-phenolic group from the caffeoyl moiety and the O-Tyr^341^ (1.835 Å) and a hydrogen bond between the H-Ser^203^ with the *m*-phenolic group (2.574 Å) on the dopamine moiety. In the lowest binding energy for *N*-*trans*-feruloyldopamine, a total of four hydrogen bonds were predicted, as follows: H-Gln^369^ and the carbonyl group (2.508 Å), O-Glu^313^ via the *m*- and *p*-phenolic group from the dopamine moiety (1.805 and 1.833 Å), and H-Asn^233^ and the *p*-phenolic group from the dopamine moiety (1.939 Å). Also, in our case, a great correlation was obtained between experimentally obtained values of *IC*_50_ and the estimated ΔG for the binding positions. Most of the predicted binding sites were already proven places of excellent inhibition with donepezil, huperzine A, tacrine and its hybrids (Gln^369^, Glu^313^, and Asn^233^) [45,46], organophosphorus compounds (Ser^203^) [47], coumarins, and phenolic acids (Tyr^341^) [2]. 

### 3.3. Antioxidant Activity

Phenolic acids and other phenolic compounds belong to natural compounds with potent antioxidant activity [6]. This activity is the consequence of their redox properties that allow them to scavenge free radicals, decompose peroxides, reduce transition metal ions, and stop chain reactions.

This study investigated antioxidant potential based on the ability to scavenge non-biological stable free radicals (DPPH^•^ and ABTS^•+^) and reduce ferric and chelate ferrous ions. By the mechanism of antioxidant action, DPPH^•^, ABTS^•+^, and FRAP assays belong to the reduction tests, which result in the transfer of hydrogen atom and/or electron from the antioxidant molecule [38]. The results obtained by these methods are presented in the Table 2. 

The antioxidant activity of tested compounds, against non-biological free radicals and ferric ion reduction, decreased in the following order: *N*-*trans*-caffeoyldopamine > *N*-*trans*-feruloyldopamine > *trans*-caffeic acid > *trans*-ferulic acid. This sequence indicates that the antioxidant activity of the test compounds is due to their hydrogen-donating ability and the number of phenolic groups. For these assays, the correlation of the number of phenolic groups and the resulting antioxidant activity was good (*r*^2^ > 0.932). The second explanation lies in the fact of proven excellent antioxidative activity of phenolic compounds with catechol arrangement (as in the case of caffeic acid and dopamine) through the stabilization of the semiquinone radical by intramolecular hydrogen bonds [48,49]. An effect that also works in favor of this is that the electron donor *o*-hydroxyl group on the aromatic ring reduces O–H bond dissociation enthalpy and thus improves antiradical activity [50]. The *o*-methoxy group has a similar effect, but not to the same extent, which is a possible explanation for why feruloyl analogs show lower activity than the corresponding caffeoyl. In addition, the acid proton appears to have little impact on the antioxidant activity, while the allylic group enhances the resonance stability of phenoxyl radical and improves the antioxidant activity [51].

**Figure 3:**
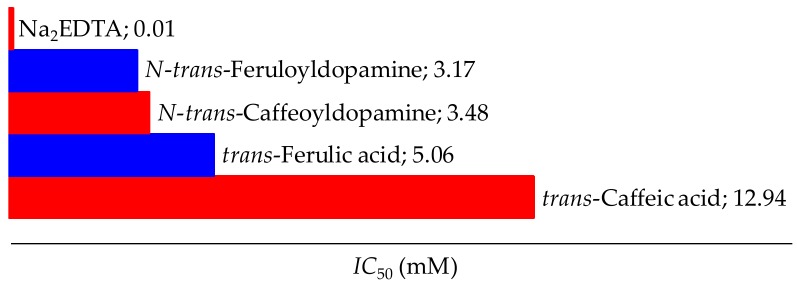
Chelation ability of tested compounds.

The direct reaction of antioxidants with free radicals is not the only mechanism by which the free radical species are inactivated. There is another mechanism, by which antioxidants do not convert free radicals into less reactive species, but rather slow down the rate of the oxidation reaction through several mechanisms [52]. The most important mechanism of secondary antioxidants is the chelation of pro-active metal ions [53]. Iron, copper, and other transition metals promote oxidation reactions by catalyzing free radical reactions. These redox active metals carry out the transfer of one electron by changing the oxidation state. By chelation, their pro-oxidative effect decreases by reducing redox potential and stabilizing their oxidation state. These chelating compounds can also sterically hinder the formation of hydroperoxide metal complexes. This effect was investigated by the method of inhibiting the formation of the purple complex between ferrous ions and ferrozine. The obtained results are presented in Figure 3.

The iron binding ability of the chelators could be related to the type of functional group used in iron chelation [54]. As both *N*-*trans*-caffeoyldopamine (3.48 μM) and *N*-*trans*-feruloyldopamine (3.17 μM) have lower *IC*_50_ values than their parent molecules, it has been shown that an additional catechol group via dopamine or the presence of the amide group increases the ability to chelate ferrous ions. But the results also suggest a low activity of all investigated compounds compared to the hexadentate Na_2_EDTA, which was used as a positive control. Because Fe^2+^ behaves as a borderline Lewis acid, it does not bind as strongly to the hard oxygen atoms of phenol ligands and the low activity of the test compounds can be attributed to the low stability of the complexes and their oxidation in the presence of O_2_ [55].

## 4. Conclusions

The results of this study indicate that *N*-*trans*-caffeoyldopamine and *N*-*trans*-feruloyldopamine fulfill the requirements to be considered potent antioxidants because they not only inhibit the radical-mediated process through reactions involving hydrogen atom and electron transfer, but also through chelating transition metals. Moreover, introducing the dopamine moiety in hydroxycinnamic acids resulted in a significant increase in AChE inhibition, which suggesting these compounds could be used as potential active ingredients of some medicines.

## Figures and Tables

**Table 1:** In vitro inhibition of acetylcholinesterase (AChE).

Compound	AChE Inhibition *IC*_50_ (μM)
*trans*-Caffeic acid	42.81 ± 1.79
*trans*-Ferulic acid	20.57 ± 0.65
*N*-*trans*-Caffeoyldopamine	19.12 ± 0.83
*N*-*trans*-Feruloyldopamine	8.52 ± 0.27
Galantamine	3.89 ± 0.10

**Table 2:** Antioxidant activity of tested compounds.

Compound	DPPH^•^ Scavenge *IC*_50_ (μM)	ABTS^•+^ Scavenge *IC*_50_ (μM)	FRAP *AAE* (μmol/mmol)
*trans*-Caffeic acid	18.86 ± 0.22	1.19 ± 0.02	526.05 ± 12.87
*trans*-Ferulic acid	19.93 ± 0.18	1.62 ± 0.01	486.80 ± 11.75
*N*-*trans*-Caffeoyldopamine	5.95 ± 0.12	0.24 ± 0.00	822.45 ± 13.53
*N*-*trans*-Feruloyldopamine	12.29 ± 0.04	0.74 ± 0.00	661.53 ± 13.51
l(+)-Ascorbic acid	1.14 ± 0.03	0.11 ± 0.00	-

DPPH^•^: 2,2-diphenyl-1-pycrylhydrazyl; ABTS: 2,2′-azinobis(3-ethylbenzothiazoline-6-sulphonic acid); FRAP: ferric-reducing antioxidant power.

## Supplementary Materials

The following are available online at http://www.mdpi.com/2218-0532/86/2/11/s1. Figure S1: MS spectra of *N*-*trans*-caffeoyldopamine, Figure S2: MS spectra of *N*-*trans*-feruloyldopamine, Figure S3: ^1^H-NMR spectra of *N*-*trans*-caffeoyldopamine, Figure S4: ^1^H-NMR spectra of *N*-*trans*-feruloyldopamine, Figure S5: ^13^C-NMR spectra of *N*-*trans*-caffeoyldopamine, Figure S6: ^13^C-NMR spectra of *N*-*trans*-feruloyldopamine.
