# Supplementary material for: Acetylcholinesterase Inhibition and Antioxidant Activity of N-trans-Caffeoyldopamine and N-trans-Feruloyldopamine
Source: Sci Pharm. 2018 Apr 4;86(2):11. doi: 10.3390/scipharm86020011 (PMC6027674; doi:10.3390/scipharm86020011)
Supplement: Supplementary file 1 [file scipharm-86-00011-s001.pdf]

Scientia Pharmaceutica

ÖSTERREICHISCHE PHARMAZEUTISCHE GESELLSCHAFT MDPI

Supplementary materials

Acetylcholinesterase inhibition and antioxidant activity of *N-trans*-caffeoyldopamine and *N-trans*-feruloyldopamine

Muamer Dizdar [1], Danijela Vidic [1], Franc Požgan [2], Bogdan Štefane [2] and Milka Maksimović [1,*]

[1] Faculty of Science, University of Sarajevo, Zmaja od Bosne 33-35, 71000 Sarajevo, Bosnia and Herzegovina
[2] Faculty of Chemistry and Chemical Technology, University of Ljubljana, Večna pot 113, 1000 Ljubljana, Slovenia
* Correspondence: mmaksimo@pmf.unsa.ba; Tel.: +387 33 279 999

1. General information

All chemicals were purchased from commercial sources and when necessary, purified following the guidelines of Armarego and Chai [28]. The monitoring of the reaction and purity of the obtained compounds was done by thin-layer chromatography (TLC) on silica gel 60 F_{254}, while the column chromatography was performed on silica gel (100–200 mesh). The visualization of the chromatograms was performed either under ultraviolet light and/or with 5% $FeCl_3$. The melting points were determined using a hot stage apparatus and are uncorrected. Infrared spectra were collected as potassium bromide (KBr) pellets in the 4000–400 cm^{-1} region with a Perkin Elmer BX Fourier-transform infrared spectrometer (Perkin Elmer, Waltham, Massachusetts, USA). The peak intensities are specified as strong (s), medium (m), or broad (br). The proton and carbon-13 nuclear magnetic resonance (^{1}H- and ^{13}C-NMR) spectra were recorded using Bruker AVANCE Ultrashield 500 plus (Bruker, Billerica, Massachusetts, USA), using deuterated dimethyl sulfoxide (DMSO-d_6) as the solvent. All shifts are given in ppm (δ) relative to tetramethylsilane and calibrated by the residual proton signal of DMSO-d_6. The coupling constants (J values) are expressed in Hz. High-resolution mass spectra (MS)were obtained by the Agilent 6224 TOF mass spectrometer (Agilent Technologies, Santa Clara, California, USA). All spectrophotometric measurements were made on a Perkin Elmer Lambda 25 UV–Vis spectrophotometer (Perkin Elmer, Norwalk, Massachusetts, USA).

2. Synthesis

Triethylamine (0.28 mL, 2 mmol) was added to a stirring solution of *trans*-hydoxycinnamic acid (2 mmol) dissolved in dimethylformamide (10 mL). The solution was cooled in an ice water bath and 2 mmol of dopamine hydrochloride was added followed by a solution of 2 mmol of (benzotriazol-1-yloxa)tris(dimethylamino)phosphonium hexafluorophosphate (BOP) in 5 mL dichloromethane. The mixture was stirred at 0 °C for 30 min and then at room temperature overnight. Dichloromethane was removed under reduced pressure and the resulting mixture was diluted with 100 mL water and extracted with ethyl acetate (3 × 30 mL). The combined organic layers were washed with HCl (3 × 15 mL, 0.5 M), $NaHCO_3$ (3 × 15 mL, 0.5 M), water (3 × 15 mL), and dried ($MgSO_4$) and evaporated under reduced pressure. The mixture was then absorbed onto SiO_2 (≈1 g, 100–200 mesh) and purified by column chromatography (ethyl acetate–hexane, 1:2→2:1) to give the crude products.

2.1. N-trans-*Caffeoyldopamine*

Yield: 258 mg, 41%; Beige solid; R_f 0.24 (EtOAc–hexane, 1:1); Melting point 182–184 °C; ν/cm^{-1} (KBr): 3450–3250 (br, s), 1653 (s), 1600 (s), 1521 (s), 1443 (s), 1372 (m), 1283 (s), 1195 (s), 1113 (s); ^{1}H-NMR (500 MHz, DMSO-d_6) δ_H: 9.34 (s, 1H), 9.11 (s, 1H), 8.75 (s, 1H), 8.64 (s, 1H), 8.01 (t, J = 5.7 Hz, 1H), 7.23 (d, J =

15.7 Hz, 1H), 6.94 (d, *J* = 2.1 Hz, 1H), 6.83 (dd, *J* = 8.2, 2.1 Hz, 1H), 6.74 (d, *J* = 8.1 Hz, 1H), 6.64 (d, *J* = 7.9 Hz, 1H), 6.60 (d, *J* = 2.1 Hz, 1H), 6.46 (dd, *J* = 8.1, 2.1 Hz, 1H), 6.32 (d, *J* = 15.7 Hz, 1H), 3.30 (q, *J* = 7.0 Hz, 2H), 2.57 (t, *J* = 7.5 Hz, 2H); ^{13}C-NMR (125 MHz, DMSO-d_6) δ_C: 165.75, 147.71, 145.99, 145.52, 144.00, 139.40, 130.74, 126.89, 120.83, 119.68, 119.08, 116.45, 116.21, 115.96, 114.26, 41.19, 35.23; HRMS (ESI+, *m*/*z*): 316.1179 [M + H]$^+$ ($C_{17}H_{18}NO_5$ requires 316.1177).

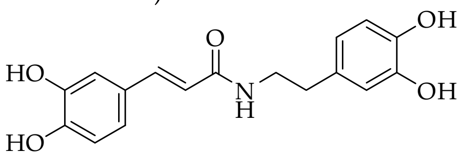

2.2. N-trans-*Feruloyldopamine*

Yield: 349 mg, 53%; Pale yellow solid; R_f 0.32 (EtOAc–hexane, 1:1); Melting point 144–145 °C; ν/cm^{-1} (KBr): 3450–3250 (br, s), 1654 (s), 1594 (s), 1516 (s), 1448 (m), 1374 (m), 1272 (s), 1210 (m), 1160 (m), 1123 (m); ^{1}H-NMR (500 MHz, DMSO-d_6) δ_H: 9.41 (s, 1H), 8.75 (s, 1H), 8.64 (s, 1H), 7.97 (t, *J* = 5.7 Hz, 1H), 7.31 (d, *J* = 15.6 Hz, 1H), 7.11 (d, *J* = 2.0 Hz, 1H), 6.98 (dd, *J* = 8.2, 2.0 Hz, 1H), 6.79 (d, *J* = 8.1 Hz, 1H), 6.64 (d, *J* = 7.9 Hz, 1H), 6.60 (d, *J* = 2.1 Hz, 1H), 6.46 (dd, *J* = 8.0, 2.1 Hz, 1H), 6.43 (d, *J* = 15.7 Hz, 1H), 3.80 (s, 3H), 3.31 (q, *J* = 6.9 Hz, 2H), 2.58 (t, *J* = 7.4 Hz, 2H); ^{13}C-NMR (125 MHz, DMSO-d_6) δ_C: 165.74, 148.68, 148.28, 145.52, 143.99, 139.30, 130.71, 126.91, 121.96, 119.67, 119.54, 116.44, 116.11, 115.96, 111.20, 55.98, 41.15, 35.18; HRMS (ESI+, *m*/*z*): 330.1336 [M + H]$^+$ ($C_{18}H_{20}NO_5$ requires 330.1340).

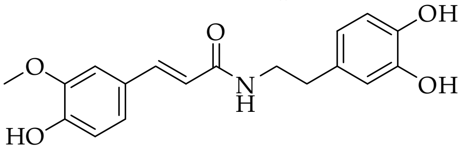

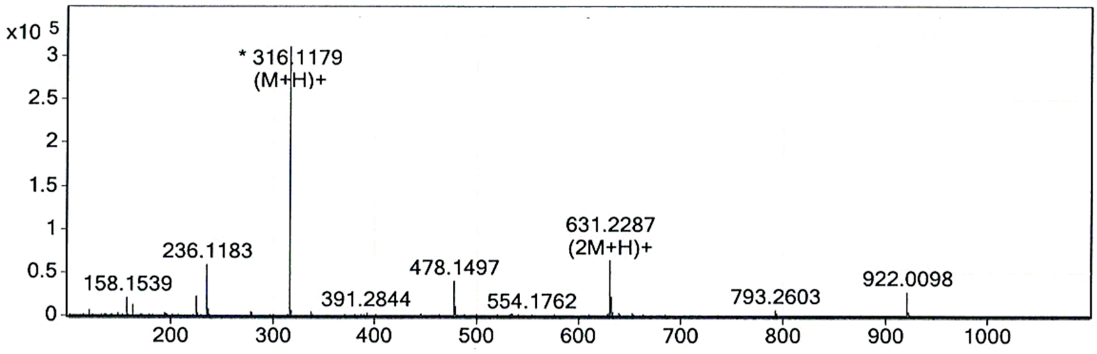

Figure S1: MS spectra of *N*-*trans*-caffeoyldopamine.

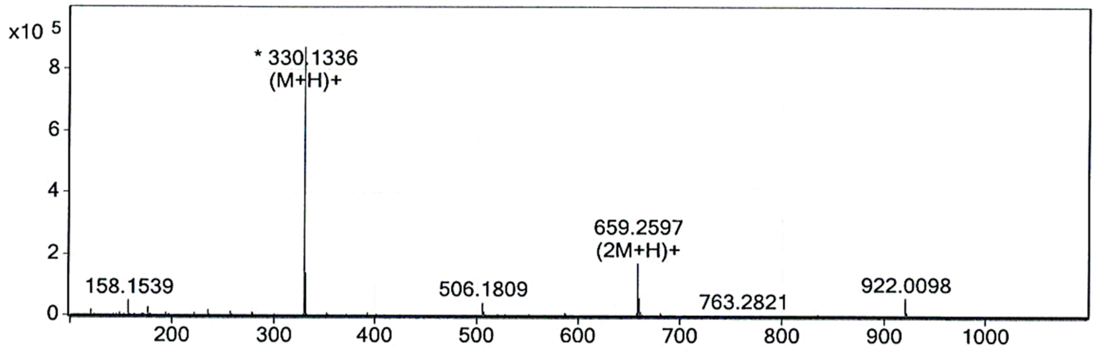

Figure S2: MS spectra of *N*-*trans*-feruloyldopamine.

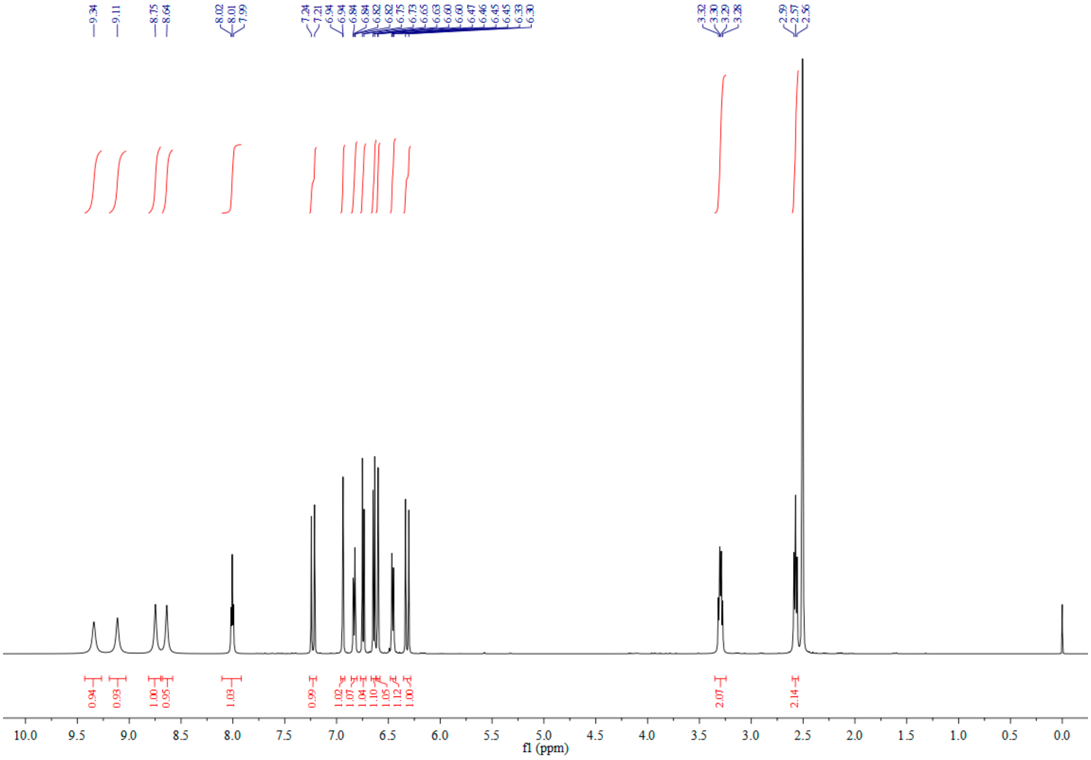

Figure S3: ^{1}H-NMR spectra of *N-trans*-caffeoyldopamine.

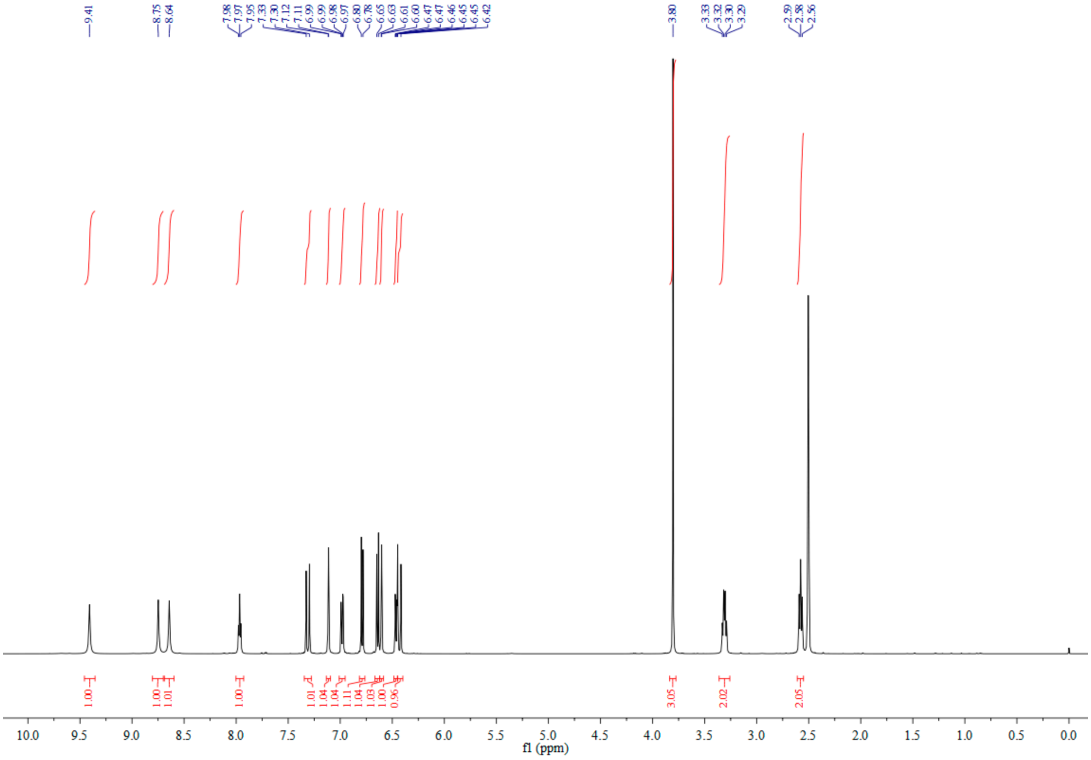

Figure S4: ^{1}H-NMR spectra of *N-trans*-feruloyldopamine.

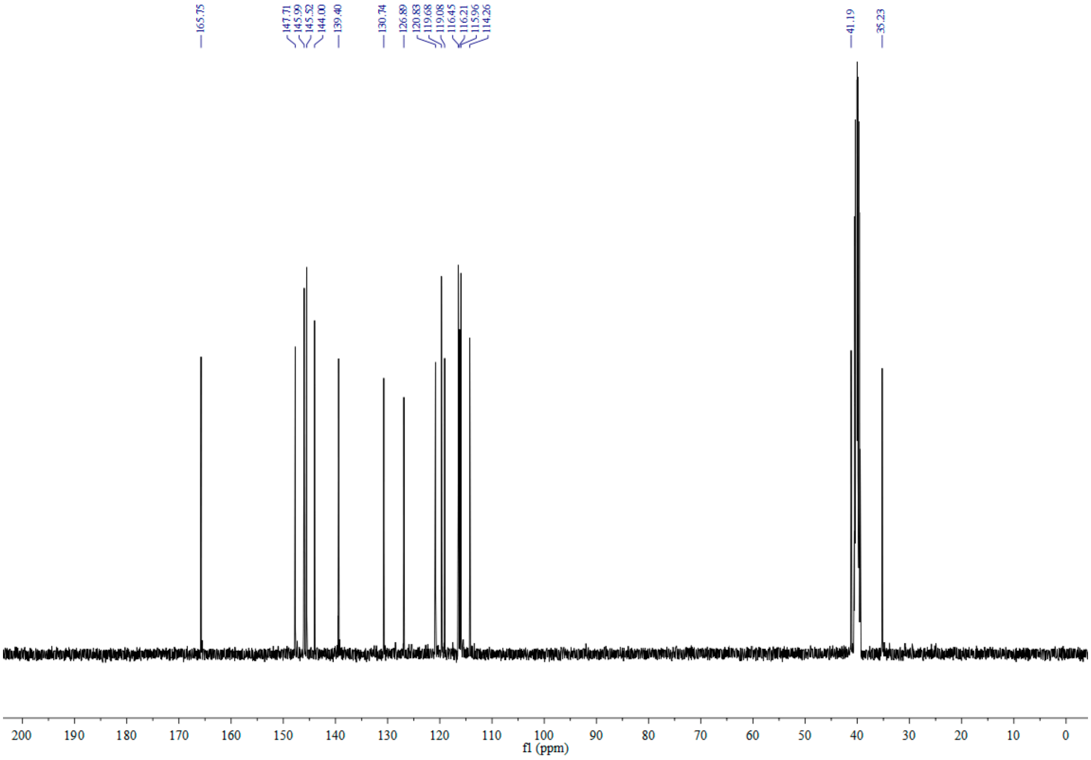

Figure S5: ^{13}C-NMR spectra of *N*- *N*-*trans*-caffeoyldopamine.

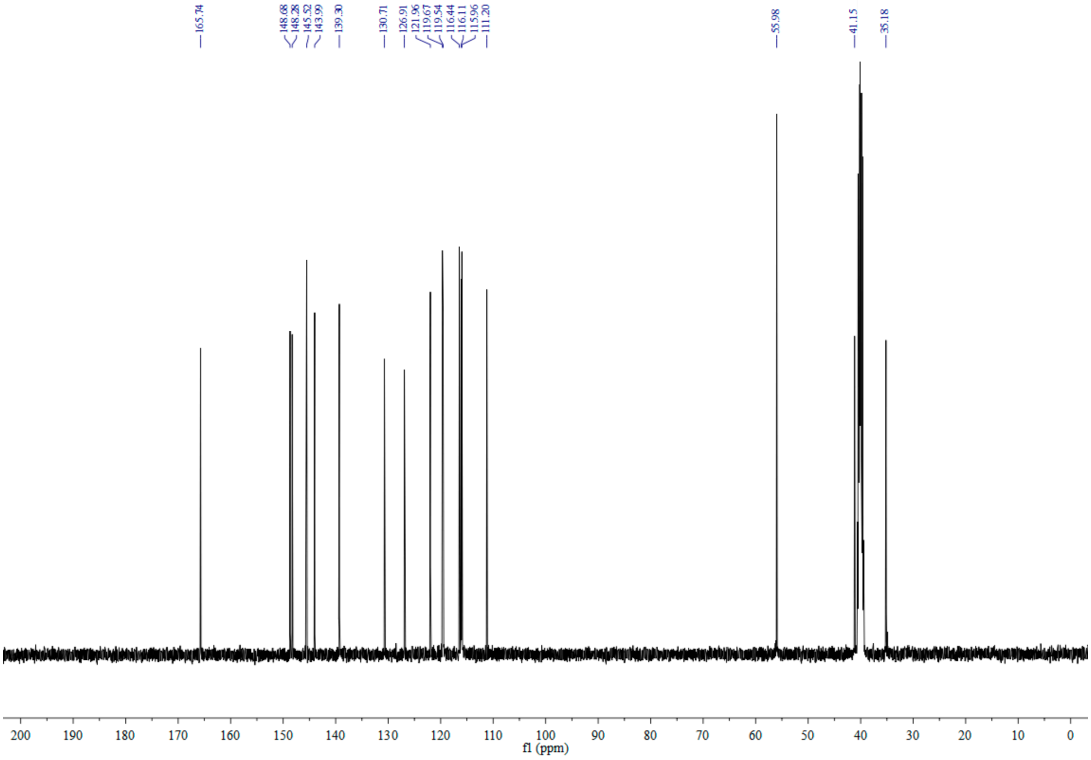

Figure S6: ^{13}C-NMR spectra of *N*-*trans*-feruloyldopamine.
